# The Comma-bacillus

**Published:** 1885-02

**Authors:** 


*The Comma-bacillus.*—The *Wiener mediz. Woch.* asserts that Koch has been able to demonstrate the transmission of the Comma-bacillus in animals and has produced the cholera in rabbits. That he has also demonstrated the non-identity between the bacilli of *cholera nostras* and Asiatic cholera.—*Italia Medica.*

